# Epidemiology and outcomes of adult (non-maternity) community-onset sepsis in Ireland: a secondary analysis of acute hospital administrative data 2020–2024

**DOI:** 10.1007/s11845-026-04504-x

**Published:** 2026-06-20

**Authors:** Nandakumar Ravichandran, Diarmuid Quinlan, Patricia Fitzpatrick, Ellen Hayes, Mick Molloy, Etimbuk Umana, Tomas Breslin, Aoife Cotter, Walter Cullen

**Affiliations:** 1 School of Medicine, University College Dublin, Dublin, Ireland; 2 Irish College of GPs, Dublin, Ireland; 3 Dept of General Practice, University College Cork, Cork, Ireland; 4 School of Public Health, Physiotherapy and Sports Science, University College Dublin, Dublin, Ireland; 5 Wexford General Hospital, Wexford, Ireland; 6 Mater Misericordiae University Hospital, Dublin, Ireland

**Keywords:** Community-onset sepsis, Epidemiology, Multimorbidity, National, Public hospitals

## Abstract

**Background:**

Community-onset sepsis accounts for most sepsis episodes, yet national-level data describing its epidemiology and outcomes remain limited in Ireland. Improved characterisation is needed to support prevention, early recognition, and service planning.

**Objectives:**

To examine the epidemiology, infection sources, comorbidity burden, and outcomes of adult (non-maternity) community-onset sepsis in Irish hospitals.

**Methods:**

We conducted a secondary analysis of Hospital In-Patient Enquiry data from all acute public hospitals in Ireland from January 2020 to December 2024. Sepsis admissions were identified using ICD-10-AM diagnostic codes. Demographic characteristics, comorbidity burden, infection source, critical care utilisation, and in-hospital mortality were analysed. Multivariable logistic regression was used to identify independent predictors of critical care admission and mortality.

**Results:**

Among 68,644 sepsis episodes, 49,268 (71.7%) were community-onset. Of these, 92.6% (*n* = 45,641) were adult (non-maternity). In-hospital mortality among community-onset was 19.2%. Mortality increased significantly with age, with episodes of individuals aged ≥ 95 years having the highest odds of death (adjOR 9.17, 95% CI 7.31–11.52) compared to those aged 25–44 years. Increasing multimorbidity was independently associated with mortality; three or more comorbidities were associated with over threefold higher odds of death (adjOR 3.24, 95% CI 2.98–3.52). Respiratory infections (adjOR 2.05) were associated with increased mortality, while urinary sources were associated with lower mortality (adjOR 0.50).

**Conclusions:**

Community-acquired sepsis represents 72% of sepsis episodes in Ireland, predominantly affects older adults with multimorbidity. Prioritising prevention, early recognition, and timely management in the community including vaccination and prompt antibiotic treatment is essential to lowering morbidity and mortality.

**Supplementary Information:**

The online version contains supplementary material available at https://doi.org/10.1007/s11845-026-04504-x.

## Introduction

 Sepsis is a life-threatening medical emergency characterised by a dysregulated host immune response to infection, leading to widespread inflammation, tissue injury, organ dysfunction [1]. Early identification and timely management are critical; however, recognition remains challenging, particularly in primary care and community settings [2]. Globally, sepsis present at hospital admission, commonly referred as Community-Onset Sepsis, accounts for more than 70% of episodes and represents a substantial public health burden [3–5].

A recent Irish population-based study examining sepsis between 2016 and 2022 reported 86,011 community-onset episodes (85.2%) and 14,930 hospital-onset sepsis episodes (14.8%). In that study, hospital-acquired sepsis was defined using a time-based criterion (infection onset ≥ 48 h after admission) [6]. While national incidence has been described, important gaps remain in understanding infection sources and multimorbidity patterns among episodes presenting with sepsis at hospital admission [7]. Improved characterisation of community-onset sepsis is needed to inform prevention strategies, support early diagnosis and management, service planning and resource allocation.

Identifying infection sources and underlying chronic disease burden is central to improving risk stratification and facilitating targeted, proactive care [8]. The use of routinely collected administrative health data for research can provide unique insights to inform decision-making and, ultimately, support better public health outcomes [9].

This study aims to examine the epidemiology, infection sources, and comorbidity patterns of adult (non-maternity) community-onset sepsis in Ireland using national hospital administrative data from 2020 to 2024. Specifically, it provides the first national characterisation integrating demographic, socioeconomic, infection-source, and comorbidity burden, and evaluates associations with key hospital outcomes including length of stay, intensive care utilisation, and in-hospital mortality.

## Methods

### Study design

This retrospective study used data from the Hospital In-Patient Enquiry (HIPE) system, a national administrative database capturing discharge records from all acute public hospitals in Ireland. All hospital discharges containing a sepsis-related diagnostic code (Supplementary file - Table 1) for adult (non-maternity) episodes between 1 January 2020 and 31 December 2024 were included. Adult (non-maternity) episodes were defined as those occurring in individuals aged 16 years or older who were not pregnant and not within 42 days postpartum. Sepsis episodes were identified using both principal and additional diagnosis fields, as sepsis may be recorded in any diagnostic position.

### Definition of community-Onset and hospital-Onset Sepsis

Because HIPE data lack reliable timestamps for infection onset, we did not use the conventional time-based definition of hospital-acquired sepsis (≥ 48 h after admission). Instead, consistent with international administrative data methodology, classification was based on the HIPE hospital-acquired diagnosis (HADx) indicator assigned directly to the relevant diagnostic codes [10–14].

A comprehensive diagnostic profile comprising general infectious diseases (Chapters A–B), localised organ-specific infections (Chapters G, J, K, L, N), systemic sepsis/septic shock (R57, R65), obstetric/neonatal infections (O85–O86, P36), healthcare-associated complications (T80–T88), and the nosocomial infection indicator (Y95) was evaluated using this flag. Community-onset sepsis is defined as episodes where the diagnostic codes establishing the sepsis event or its underlying infection profile were recorded as present on admission, lacking a hospital-acquired flag (HADx = 0). Hospital-onset sepsis is defined as episodes where these diagnostic or complicating codes were explicitly flagged by clinical coders as having developed during the hospital stay (HADx = 1). While individual administrative codes alone do not establish clinical causality, tracking the presence of HADx = 1 across this specific diagnostic cluster provides a highly plausible, standardised signal linking the onset of the sepsis cascade to healthcare procedures, device or hospital exposures. This approach aligns with methodologies used in Centers for Disease Control and Prevention (CDC) National Healthcare Safety Network (NHSN) definitions and the European Centre for Disease Prevention and Control (ECDC) criteria [15, 16].

### Data collected

The following variables were extracted from the HIPE dataset for adult (non-maternity) community-onset episodes: year of discharge, sex, age group, length of stay in hospital, length of stay in critical care (ICU or coronary care unit), all additional diagnostic codes, General Medical Service (GMS) status, and any procedures undergone during admission. Up to 30 diagnostic codes were available for each admission and were classified using ICD-10-AM. The 10th edition was used for records from 2020 to 2023 and the 12th edition for 2024. GMS eligibility provides free access to publicly funded primary and secondary healthcare services in Ireland and was used as an indicator of health service utilization and financial protection.

Episodes of individuals were classified into age groups based on life stages: young adults (16–24 years), early adults (25–44 years), middle-aged adults (45–64 years), young-old adults (65–74 years), middle-old adults (75–84 years), old-old adults (85–94 years), and centenarians (95 + years).

For community-onset sepsis admissions, diagnostic codes were matched to different sources of infection and 17 Charlson comorbidity categories and number of comorbidities variable were calculated from other diagnoses of sepsis episodes [16, 17] (Supplementary file - Tables 2 and 3). The mapping was carried out in Jupyter notebook Python 3.12.

### Statistical analysis

The final logistic regression models for critical care admission and in-hospital mortality were performed on the adult non-maternity cohort (*n* = 45,641). Age, sex, and GMS eligibility were included in all multivariable models as a priori confounders regardless of statistical significance for adult (non-maternity) (≥ 16 years) community-onset sepsis episodes [18]. All infection source categories were initially entered into the model because of their potential clinical relevance. Due to the large number of infection source variables, backward stepwise elimination was applied to remove variables with *p* > 0.05 while retaining age, sex, and GMS status in the final model. Adjusted odds ratios (ORs) with 95% confidence intervals (CIs) were reported. Statistical analyses were performed using SPSS version 29. Where fewer than 10 outcome events were observed within a category, categories were grouped or analyses were restricted to univariable logistic regression.

### Ethical approval

Ethical approval was obtained from the University College Dublin Human Research Ethics Committee (288-LS-LRSD-25-Ravichandran-Cullen). The HIPE dataset is fully anonymised and non-identifiable; therefore, informed consent was not required. All procedures were conducted in accordance with the Declaration of Helsinki (1975). This study is reported in accordance with the Strengthening the Reporting of Observational Studies in Epidemiology (STROBE) guidelines.

## Results

### Trends in community- and hospital-onset sepsis (2020–2024)

**Fig. 1:**
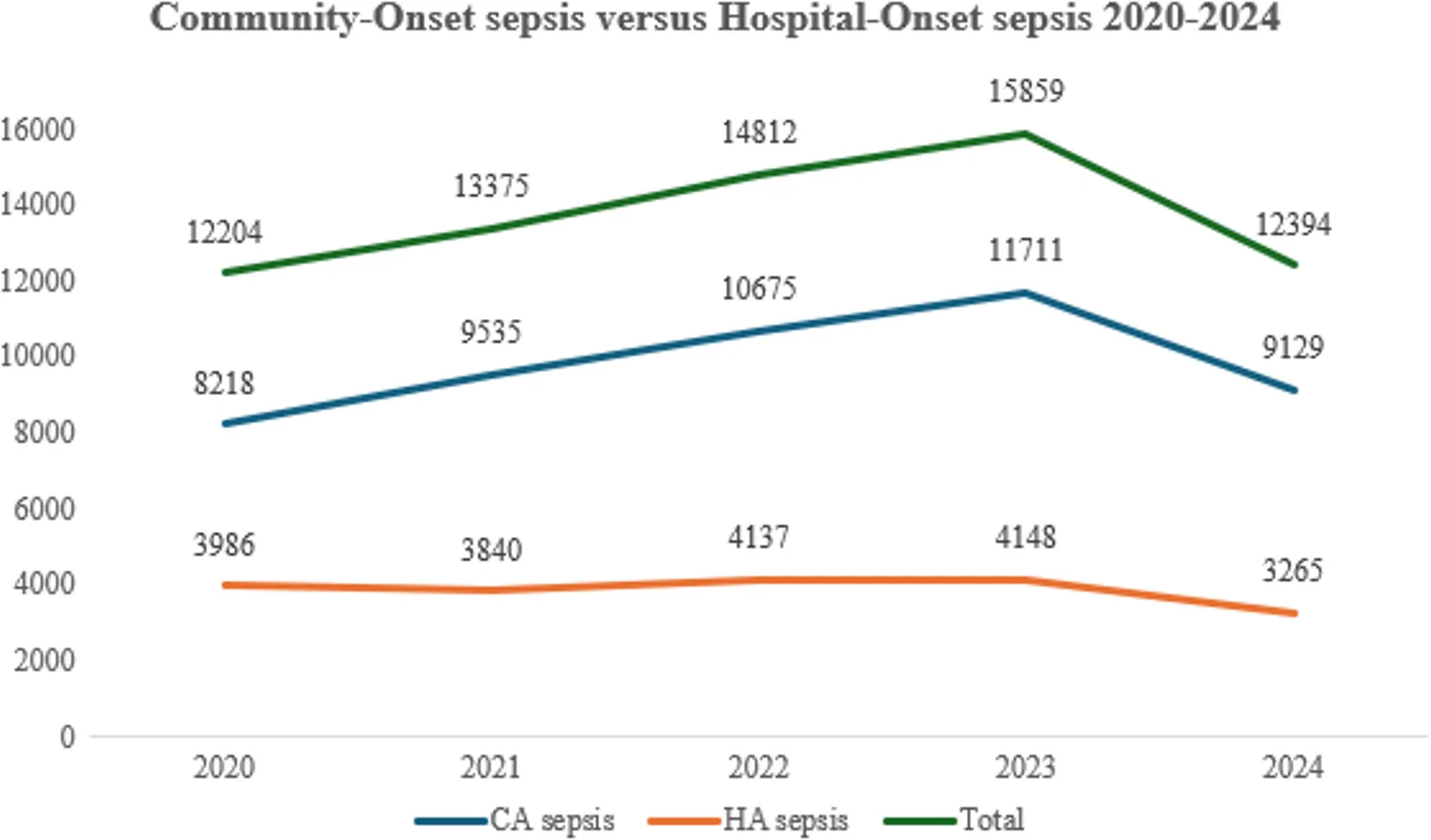
Distribution of community-onset and hospital-onset sepsis during the period 2020–2024

Between 2020 and 2024, a total of 68,644 sepsis admissions were identified across all age groups. Of these, 71.7% (*n* = 49,268) were classified as community-onset sepsis, and 28.3% (*n* = 19,376) as hospital-onset sepsis (Fig. 1). The number of community-onset sepsis episodes increased annually from 2020 (*n* = 8,218) to a peak in 2023 (*n* = 11,711), followed by a decline in 2024 (*n* = 9,129). In contrast, hospital-onset sepsis episodes remained relatively stable between 2020 (*n* = 3,986) and 2023 (*n* = 4,148), before decreasing in 2024 (*n* = 3,265).

Among community-onset sepsis episodes, 92.6% (*n* = 45,641) were adult (non-maternity).

### Demographic characteristics and admission pathway

Among adult (non-maternity) community-onset sepsis episodes, 54.7% (*n* = 24,951) occurred in males and 45.3% (*n* = 20,690) in females. Of these 45,641 episodes, 71.6% occurred in individuals aged 65 years or older. The 75–84-year age group represented the largest proportion (29.3%, *n* = 13,386).

Two-thirds of episodes (69.8%, *n* = 31,814) were eligible for the General Medical Services scheme, providing free access to publicly funded primary and secondary healthcare services. Most episodes were emergency admissions (95.7%, *n* = 43,696), reflecting acute presentation (Table 1).

**Table 1:** Characteristics of adult (non-maternity) community-onset sepsis episodes (*n* = 45,641)

Demographic Variables	Number of episodes (*n* = 45,641) *n* (%)
Sex	
Male	24,951 (54.7)
Female	20,690 (45.3)
Age group	
16–24 years	617 (1.4)
25–44 years	2707 (5.9)
45–64 years	9641 (21.1)
65–74 years	10,663 (23.4)
75–84 years	13,386 (29.3)
85–94 years	7991 (17.5)
95 + years	636 (1.4)
GMS status	
GMS	31,814 (69.8)
Non-GMS	13,784 (30.2)
Type of admission	
Elective	1945 (4.3)
Emergency	43,696 (95.7)
Source of infection^1^	
Respiratory	17,289 (37.9)
Urinary	16,728 (36.7)
Abdominal	7559 (16.6)
Skin	4947 (10.8)
Cardiac/Vascular	1177 (2.6)
Bone/joint	843 (1.8)
Nervous	179 (0.4)
Number of comorbidities	
0	15,857 (34.7)
1	15,624 (34.2)
2	9664 (21.2)
3	3370 (7.4)
4+	1126 (2.4)
Discharge status	
Home	25,517 (56.6)
Long stay accommodation	6730 (14.9)
Transfer to hospital	4211 (9.3)
Died	8659 (19.2)
Length of stay (in days)	
Median (range)	9 (0.5–459)
Length of stay in critical care (in days)	
(*n* = 9750, 21.4%)	
Median (range)	4 (0.5–133)

^1^Episodes may have more than one documented infection source; categories are not mutually exclusive

### Infection Sources and Comorbid Burden

Episodes could have more than one infection source or comorbidity, not mutually exclusive events. Respiratory and urinary tract infections were the most common sources of infection, followed by abdominal and skin/soft tissue infections (Table 1). The burden of comorbidity was substantial: 65.3% episodes had at least one recorded comorbidity, with 9.8% (*n* = 4,496) had three or more comorbidities.

### Clinical Outcomes

The median hospital length of stay was 9.0 days (range: 0.5–459 days). For 21.4% (*n* = 9,750) requiring critical care, the median ICU length of stay was 4 days (range: 0.5–133), indicating that while many episodes were managed in general wards, a subset of episodes required prolonged intensive support. In-hospital mortality rate among community-onset episodes was 19.2% (*n* = 8,659) (Table 1).

The clinical outcomes of adult (non-maternity) community-onset sepsis episodes are presented in Table 2.

**Table 2:** Critical care admission and mortality by age group

Adult (non-maternity)	Critical care admission (*N* = 9750) *n* (%)	Mortality (*N* = 8659) *n* (%)	Number of episodes (*N* = 45641) *n* (%)
16–24 years	157 (1.6)	22 (0.3)	617 (1.4)
25–44 years	751 (7.7)	181 (2.1)	2707 (5.9)
45–64 years	2682 (27.5)	1123 (13.0)	9641 (21.1)
65–74 years	2684 (27.5)	1744 (20.1)	10,663 (23.4)
75–84 years	2573 (26.4)	2913 (33.6)	13,386 (29.3)
85–94 years	875 (9.0)	2417 (27.9)	7991 (17.5)
95 + years	28 (0.3)	259 (2.9)	636 (1.4)

### Predictors of Critical care admission among adult (non-maternity) community-onset sepsis episodes (*n* = 9750 admissions)

Sex was not significantly associated with critical care admission. Compared with adults aged 25–44 years, older age groups had progressively lower odds of critical care admission (*p* < 0.001). GMS episodes had lower odds of critical care admission (*p* < 0.001). The source of infection was also a major predictor. Several infection sources were associated with increased odds of critical care admission, particularly respiratory and nervous system infections. Compared to those with no comorbidity, the critical care admission increased two-fold for episodes with three or more comorbidities (adjOR = 1.94, 95% CI 1.79–2.10, *p* < 0.001) (Table 3).

**Table 3:** Multivariable regression model of critical care admission (*n* = 9750 admissions)

Variables	OR [95% CI]	*p*-value	Adjusted OR [95% CI]	adjusted *p*-value
Female sex	0.94 [0.90–0.98]	0.004	0.99 [0.95–1.04]	0.796
GMS status	0.74 [0.71–0.78]	< 0.001	0.77 [0.74–0.82]	< 0.001
Age group^2^		< 0.001		< 0.001
16–24 years	0.89 [0.73–1.09]		0.93 [0.76–1.15]	
45–64 years	1.00 [0.91–1.10]		0.94 [0.85–1.04]	
65–74 years	0.88 [0.80–0.96]		0.80 [0.73–0.88]	
75–84 years	0.62 [0.56–0.68]		0.57 [0.51–0.63]	
85 + years	0.30 [0.27–0.34]		0.28 [0.25–0.31]	
Infection source: Respiratory	1.85 [1.77–1.94]	< 0.001	2.01 [1.92–2.12]	< 0.001
Infection source: Urinary	0.48 [0.45–0.50]	< 0.001	0.69 [0.65–0.73]	< 0.001
Infection source: Abdominal	1.78 [1.69–1.88]	< 0.001	1.91 [1.80–2.02]	< 0.001
Infection source: Skin	1.28 [1.20–1.38]	< 0.001	1.40 [1.30–1.50]	< 0.001
Infection source: Nervous	3.35 [2.50–4.50]	< 0.001	2.90 [2.13–3.96]	< 0.001
Infection source: Cardiac/Vascular	1.88 [1.66–2.13]	< 0.001	1.60 [1.41–1.82]	< 0.001
Number of comorbidities^3^		< 0.001		< 0.001
One	1.15 [1.08–1.21]		1.13 [1.06–1.19]	
Two	1.49 [1.40–1.58]		1.37 [1.28–1.46]	
Three or more	2.15 [1.99–2.31]		1.94 [1.79–2.10]	

^2^Reference: 25–44 years

^3^Reference: No comorbidity

Reference for each infection source: Absence of that specific documented infection source (e.g., ‘No Respiratory Infection’)

### Predictors of mortality (*n* = 8659 deaths)

Female sex was associated with higher mortality compared with males (adjOR 1.11, 95% CI 1.06–1.17, *p* < 0.001). Age showed a strong graded association with mortality compared with the 25–44-year reference group (*p* < 0.001). Respiratory (adjOR 2.05) and Skin (adjOR 1.36) associated with higher odds of mortality (*p* < 0.001). In contrast, urinary source of infection was associated with lower odds of death (adjOR 0.50 95% CI 0.47–0.53, *p* < 0.001). Compared to those with no comorbidity, the odds of death increased three-fold for episodes with three or more comorbidities (adjOR = 3.24, 95% CI 2.98–3.52, *p* < 0.001) (Table 4).

**Table 4:** Multivariable regression model of mortality (*n* = 8659 deaths)

Variables	OR [95% CI]	*p*-value	Adjusted OR [95% CI]	adjusted *p*-value
Female sex	1.08 [1.03–1.13]	0.002	1.11 [1.06–1.17]	< 0.001
GMS status	1.36 [1.29–1.44]	< 0.001	1.03 [0.97–1.09]	0.341
Age group^2^		< 0.001		< 0.001
16–24 years	0.52 [0.33–0.81]		0.63 [0.40–0.99]	
45–64 years	1.84 [1.56–2.17]		1.58 [1.34–1.86]	
65–74 years	2.73 [2.33–3.20]		2.26 [1.92–2.66]	
75–84 years	3.88 [3.32–4.54]		3.35 [2.85–3.93]	
85–94 years	6.05 [5.17–7.09]		5.60 [4.76–6.59]	
95 + years	9.59 [7.71–11.93]		9.17 [7.31–11.52]	
Infection source: Respiratory	2.81 [2.68–2.95]	< 0.001	2.05 [1.95–2.16]	< 0.001
Infection source: Urinary	0.47 [0.45–0.50]	< 0.001	0.50 [0.47–0.53]	< 0.001
Infection source: Abdominal	1.03 [0.97–1.09]	0.405	1.07 [1.00-1.15]	0.040
Infection source: Skin	1.51 [1.41–1.62]	< 0.001	1.36 [1.26–1.47]	< 0.001
Infection source: Nervous	1.08 [0.75–1.55]	0.697	1.65 [1.12–2.43]	0.011
Infection source: Cardiac	0.66 [0.56–0.78]	< 0.001	0.78 [0.65–0.93]	0.006
Infection source: Bone/Joint	0.66 [0.54–0.81]	< 0.001	0.67 [0.54–0.82]	< 0.001
Number of comorbidities^3^		< 0.001		< 0.001
One	1.69 [1.59–1.80]		1.50 [1.41–1.60]	
Two	2.39 [2.24–2.56]		2.16 [2.01–2.32]	
Three or more	3.90 [3.61–4.22]		3.24 [2.98–3.52]	

^2^Reference: 25–44 years

^3^Reference: No comorbidity

Reference for each infection source: Absence of that specific documented infection source (e.g., ‘No Respiratory Infection’)

## Discussion

This study provides the first national characterisation of adult (non-maternity) community-onset sepsis in Ireland, demonstrating that the majority of sepsis admissions originate in the community and are concentrated among older adults with substantial multimorbidity.

The predominance of community-onset sepsis (72% of all sepsis admissions) aligns with international estimates, reinforcing that sepsis is largely a condition presenting prior to hospitalisation rather than acquired within it. The high proportion of emergency admissions further reflects the acute and often advanced stage at presentation, highlighting ongoing challenges in early recognition and timely intervention in community settings. Respiratory and urinary tract infections were the leading sources of infection, consistent with international literature identifying pneumonia and urosepsis as the most common drivers of sepsis [19, 20].

Older adults accounted for higher proportion of community-onset sepsis episodes, with incidence rising sharply among episodes of individuals aged 65 years and older. This aligns with established evidence linking immuno-senescence, frailty, and multimorbidity with increased susceptibility to severe infection and adverse outcomes [21–23]. The strong association between multimorbidity and mortality in this study supports the concept that sepsis frequently occurs in the context of advanced underlying illness. These findings suggest that delayed presentation, atypical symptoms, and treatment limitations or delays may contribute to poorer outcomes in frail populations, underscoring the importance of proactive chronic disease management, vaccination, and anticipatory care planning and proactive infection prevention strategies in high-risk populations [24].

Notably, while the oldest age groups experienced the highest mortality, they were significantly less likely to be admitted to critical care. This pattern suggests that escalation decisions may be influenced by age, frailty, or perceived prognosis, as described in previous studies. It may also reflect appropriate clinical decision-making based on patient preferences or treatment limitations; however, it raises important questions regarding equity of access to intensive care and warrants further investigation.

The observed association between female sex and higher in-hospital mortality is consistent with existing literature, although evidence on sex differences in sepsis outcomes remains mixed [25]. This finding may reflect residual confounding or differences in age distribution, frailty, or healthcare utilisation not captured within administrative datasets [25]. Although GMS eligibility was associated with higher mortality in unadjusted analyses, this effect was not independent after adjustment. This suggests that socioeconomic disparities in sepsis outcomes may be largely mediated through differences in age and comorbidity burden, rather than direct effects of healthcare coverage itself.

Given the general practitioners (GPs) and GP-out of hours represent the first point of contact for most patients, the findings underscore the critical role of primary care in early recognition, risk stratification, and timely referral [26]. In Ireland, general practice delivers over 21 million consultations annually, with a further 1.5 million consultations occurring through GP-out of hours services [27]. This level of contact provides substantial opportunity for sepsis prevention, early recognition, timely escalation, and effective safety-netting in the community are essential to reducing sepsis morbidity and mortality.

### Implications for research, policy and practice

The predominance of community-onset sepsis highlights the need to strengthen early recognition and escalation pathways in primary care, GP out-of-hours services, emergency medical services, and community nursing. Given the strong associations with older age, multimorbidity, common infection sources such as respiratory and urinary tract infections and GMS eligibility, targeted risk stratification strategies in community settings may support earlier identification and referral.

Preventive strategies should prioritise vaccination uptake, chronic disease optimisation, antimicrobial stewardship, and proactive monitoring of high-risk populations. Integration of administrative sepsis data with national infectious disease surveillance systems may further enhance early warning capacity and inform regional resource allocation.

### Strengths and limitations

This study uses a large, national administrative dataset capturing all discharges from acute public hospitals in Ireland, enhancing generalisability and minimising selection bias. The availability of up to 30 diagnostic codes per admission enabled detailed characterisation of infection sources and multimorbidity burden. The use of hospital-acquired diagnostic flags allowed differentiation between community- and hospital-onset sepsis using a consistent national coding framework.

Findings are subject to the inherent limitations of administrative data, including potential misclassification and reliance on documentation quality. Episodes should be interpreted as sepsis-coded admissions rather than clinically reviewed and confirmed Sepsis-3 cases. HIPE does not have clinical information including physiological, microbiological or laboratory markers, timing of therapeutic interventions, which limits the assessment of clinical pathways and severity of the disease in this population. The classification of hospital-onset and community-onset sepsis relied on administrative hospital-acquired diagnostic flags associated with infection diagnoses rather than precise clinical timestamps for sepsis onset. As a result, episodes in which infection was present on admission, but organ dysfunction and sepsis developed later during hospitalisation, may have been classified as community-onset. This approach may therefore have overestimated the burden of community-onset sepsis and underestimated hospital-onset sepsis. Similarly, some hospital-acquired infections may not have been fully captured through coding alone.

The dataset also captured sepsis episodes rather than individual patients and therefore could not account for repeat admissions or multiple sepsis episodes occurring in the same patient over time. In addition, reliance on hospital discharge data may underestimate the true burden of community-onset sepsis nationally, as individuals who deteriorated and died before hospital presentation, or who experienced severe infection managed exclusively in community settings, would not have been captured. No private hospitals are included in HIPE data, so this data reflects public hospitals only. In addition, paediatrics and pregnancy-associated sepsis episodes were excluded from the multivariable analysis to focus on the general adult (non-maternity) population (*n* = 45,641). These groups require separate analysis as their clinical pathways and risk profiles differ substantially, which will be reported in a separate paper.

## Conclusion

Community-onset sepsis in Ireland predominantly affects older adults with multimorbidity and most commonly arises from respiratory and urinary tract infections. The odds of mortality among adult (non-maternity) community episodes increased with age and comorbidity burden.

## Electronic Supplementary Material

Below is the link to the electronic supplementary material.

Supplementary file1
