# Supplementary material for: Epidemiology and outcomes of adult (non-maternity) community-onset sepsis in Ireland: a secondary analysis of acute hospital administrative data 2020–2024
Source: Ir J Med Sci. 2026 Jun 20;195(5):3205–12. doi: 10.1007/s11845-026-04504-x (PMC13740032; doi:10.1007/s11845-026-04504-x)
Supplement: Supplementary file 1 [file 11845_2026_4504_MOESM1_ESM.docx]

**Supplementary file**

**Table 1. Sepsis-related diagnostic code**

| **Code description** | **ICD-10-AM 10th** | **ICD-10-AM 12th** |
| --- | --- | --- |
| Salmonella sepsis | A02.1 | A02.1 |
| Anthrax sepsis | A22.7 | A22.7 |
| Erysipelothrix sepsis | A26.7 | A26.7 |
| Listerial sepsis | A32.7 | A32.7 |
| Streptococcal sepsis | A40 | A40 |
| Sepsis due to Streptococcus, group A | A40.0 | A40.0 |
| Sepsis due to Streptococcus, group B | A40.1 | A40.1 |
| Sepsis due to Streptococcus, group D and Enterococcus | A40.2 | A40.21, A40.22 |
| Sepsis due to Streptococcus pneumoniae | A40.3 | A40.3 |
| Other streptococcal sepsis | A40.8 | A40.8 |
| Streptococcal sepsis, unspecified | A40.9 | A40.9 |
| Other sepsis | A41 | A41 |
| Sepsis due to Staphylococcus aureus | A41.0 | A41.0 |
| Sepsis due to other specified staphylococcus | A41.1 | A41.1 |
| Sepsis due to unspecified staphylococcus | A41.2 | A41.2 |
| Sepsis due to Haemophilus influenzae | A41.3 | A41.3 |
| Sepsis due to anaerobes | A41.4 | A41.4 |
| Sepsis due to other and unspecified Gram-negative organisms | A41.5 | A41.5 |
| Sepsis due to unspecified Gram-negative organisms | A41.50 | A41.50 |
| Sepsis due to Escherichia coli [E. coli] | A41.51 | A41.51 |
| Sepsis due to Pseudomonas | A41.52 | A41.52 |
| Sepsis due to other Gram-negative organisms | A41.58 | A41.58 |
| Sepsis due to Enterococcus | No specific code available. | A40.22 |
| Other specified sepsis | A41.8 | A41.8 |
| Sepsis, unspecified | A41.9 | A41.9 |
| Actinomycotic sepsis | A42.7 | A42.7 |
| Meningococcal sepsis | A39.2 , A39.3, A39.4 | A39.7 |
| Septicaemic plague | A20.7 | A20.7 |
| Toxic shock syndrome | A48.3 | A48.3 |
| Candidal sepsis | B37.7 | B37.7 |
| Puerperal sepsis | O85 | O85 |
| Bacterial sepsis of newborn | P36 | P36 |
| Severe sepsis | R65.1 | Code removed 12th edition. |
| Septic shock | R57.2 | R57.2 |

**Table 2. Multimorbidity types and mapped associated codes**

| **Chronic Category** | **ICD-10-AM Codes (HIPE)** |
| --- | --- |
| **Myocardial Infarct** | 410, 412, I21, I22, I23, I24, I25.2 |
| **Congestive Heart Failure** | 398.91, 402.01, 402.11, 402.91, 404.01, 404.03, 404.11, 404.13, 404.91, 404.93, 425.4, 425.9, 428, I09.9, I11.0, I13.0, I13.2, I25.5, I42.0, I42.5, I42.9, I43, I50, P29.0 |
| **Peripheral Vascular Disease** | 093.0, 437.3, 440, 441, 443.1, 443.9, 447.1, 557.1, 557.9, V43.4, 785.4, I70, I71, I73.1, I73.8, I73.9, I74, I77.1, I79.0, I79.2, K55.1, K55.8, K55.9, Z95.8, Z95.9 |
| **Cerebrovascular Disease** | 362.34, 430, 431, 432, 433, 434, 435, 436, 437, 438, G45, G46, I60, I61, I62, I63, I64, I65, I66, I67, I68, I69, H34.0 |
| **Dementia** | 290, 294.1, 331.2, F00, F01, F02, F03, F05.1, G30 |
| **Chronic Pulmonary Disease** | 416.8, 416.9, 490, 491, 492, 493, 494, 495, 496, 505, 506.4, 508.1, 508.8, I27.8, I27.9, J40, J41, J42, J43, J44, J45, J46, J47, J60, J61, J62, J63, J64, J65, J66, J67, J68.4, J70.1, J70.3 |
| **Rheumatic Disease** | 446.5, 710.0, 710.1, 710.4, 714.0, 714.1, 714.2, 714.81, 725, D86, M05, M06, M30, M31.5, M32, M33, M34, M35.1, M35.3, M36.0 |
| **Peptic Ulcer Disease** | 531, 532, 533, 534, K25, K26, K27, K28 |
| **Mild Liver Disease** | 070.22, 070.23, 070.32, 070.33, 070.44, 070.54, 070.6, 070.9, 570, 571, 571.2, 571.4, 571.5, 571.6, 573.3, 573.4, 573.8, 573.9, V42.7, B18, K70.0, K70.1, K70.2, K70.3, K70.9, K71.3, K71.4, K71.5, K71.7, K73, K74, K76.0, K76.2, K76.3, K76.4, K76.8, K76.9, Z94.4 |
| **Diabetes (without complications)** | 250.0, 250.1, 250.2, 250.3, 250.7, E10.0, E10.1, E10.6, E10.8, E10.9, E11.0, E11.1, E11.6, E11.8, E11.9, E12.0, E12.1, E12.6, E12.8, E12.9, E13.0, E13.1, E13.6, E13.8, E13.9, E14.0, E14.1, E14.6, E14.8, E14.9 |
| **Diabetes (Complex)** | 250.4, 250.5, 250.6, E10.2, E10.3, E10.4, E10.5, E10.7, E11.2, E11.3, E11.4, E11.5, E11.7, E12.2, E12.3, E12.4, E12.5, E12.7, E13.2, E13.3, E13.4, E13.5, E13.7, E14.2, E14.3, E14.4, E14.5, E14.7 |
| **Hemiplegia or Paraplegia** | 334.1, 342, 343, 344.0, 344.1, 344.6, 344.9, G04.1, G11.4, G80.1, G80.2, G81, G82, G83.0, G83.1, G83.2, G83.3, G83.4, G83.9 |
| **Renal Disease** | 403.01, 403.11, 403.91, 404.02, 404.03, 404.12, 404.13, 404.92, 404.93, 582, 583, 585, 586, 588, V42.0, V45.1, V56, I12.0, I13.1, N03.2, N03.3, N03.4, N03.5, N03.6, N03.7, N05.2, N05.3, N05.4, N05.5, N05.6, N05.7, N18, N19, N25.0, Z49.0, Z49.1, Z49.2, Z94.0, Z99.2 |
| **Any Malignancy** | 140–149, 150–159, 160–169, 170–176, 177–180, 238.6, C00–C14, C15–C26, C30–C39, C40–C49, C50–C58, C60–C76, C81–C97 |
| **Severe Liver Disease** | 456.0, 456.1, 456.2, 572.2, 572.3, 572.4, 572.5, 572.6, 572.7, 572.8, I85.0, I85.9, I86.4, I98.2, K70.4, K71.1, K72.1, K72.9, K76.5, K76.6, K76.7 |
| **Metastatic Tumour** | 196, 197, 198, 199, C77, C78, C79, C80 |
| **HIV/AIDS** | 042, 043, 044, B20, B21, B22, B23, B24, Z21 |

**Table 3. Infection Source Categories and ICD codes**

| **Source Category** | **ICD Codes (HIPE)** |
| --- | --- |
| **Respiratory** | 460, 461, 462, 463, 464, 465, 480, 481, 482, 483, 484, 485, 486, 487, 488, 490, 491, 492, 493, 494, 495, 496, 507.0, 507.8, 518.6, J00, J01, J02, J03, J04, J05, J06, J10, J11, J12, J13, J14, J15, J16, J17, J18, J20, J21, J22, J44, J69, J85, J86 |
| **Urinary** | 590, 590.1, 591, 592, 593, 595, 596, 599.0, N10, N11, N12, N13.6, N15, N30, N34, N39.0, R31, R33 |
| **Abdominal** | 540, 541, 542, 543, 560, 561, 562, 563, 564, 565, 566, 567, 568, 569, 574, 575, 576, A04.7, A09, K35, K36, K37, K52.9, K56, K57, K61, K63, K65, K66, K75.0, K80, K81, K82, K83 |
| **Skin / Soft Tissue** | 680, 681, 682, 683, 684, 685, 686, 707, L00, L01, L02, L03, L04, L05, L08, L08.9, L89, M72.6, I83.2 |
| **Nervous System** | 320, 321, 322, 323, 324, 325, 326, 327, 328, 329, G00, G01, G02, G03, G04, G05, G06 |
| **Cardiac / Vascular** | 391, 392, 393, 394, 395, 396, 397, 398, 420, 421, 422, 423, 424, 425, 426, 427, 428, 429, I30, I31, I32, I33, I38, I39 |
| **Bone / Joint** | 711, 730, 731, 732, 733, M00, M01, M46, M86 |
